# Association between Schizophrenia and Urinary Calculi: A Population-Based Case-Control Study

**DOI:** 10.1371/journal.pone.0056942

**Published:** 2013-03-07

**Authors:** Shih-Ping Liu, Ching-Chun Lin, Herng-Ching Lin, Yi-Hua Chen, Hong-Jeng Yu

**Affiliations:** 1 Department of Urology, National Taiwan University Hospital, Taipei, Taiwan; 2 Graduate Institute of Biomedical Informatics, College of Medical Science and Technology, Taipei Medical University, Taipei, Taiwan; 3 School of Health Care Administration, College of Public Health and Nutrition, Taipei Medical University, Taipei, Taiwan; 4 School of Public Health, College of Public Health and Nutrition, Taipei Medical University, Taipei, Taiwan; 5 Division of Urology, Department of Surgery, Far Eastern Memorial Hosital, New Taipei City, Taiwan; Baylor College of Medicine, United States of America

## Abstract

**Background:**

People with schizophrenia have been demonstrated to have higher overall morbidity and all-cause mortality rates from general medical conditions. However, little attention has been given to the urinary system of people with schizophrenia. As no direct evidence has been reported demonstrating a link between schizophrenia and urinary calculi, this study utilized a population-based case-control study design to investigate the possibility of an association between schizophrenia and the occurrence of urinary calculi.

**Method:**

This study used data from the Taiwan Longitudinal Health Insurance Database. Cases consisted of 53,965 urinary calculi patients newly diagnosed between 2002 and 2008. In total, 269,825 controls were randomly selected and matched with the cases in terms of age and sex. Each person was traced to discern whether he had previously received a diagnosis of schizophrenia. Conditional logistic regression models were performed for the analysis.

**Results:**

A total of 3,119 (1.0%) subjects had been diagnosed with schizophrenia prior to the index date. This included 0.7% of the patients with urinary calculi, and 1.0% of the controls. A prior diagnosis of schizophrenia was independently associated with a 30% decrease (95% CI = 0.62–0.76) in the occurrence of urinary calculi. The reduction was even more remarkable in males (38%, 95% CI = 0.55–0.71) and in elder individuals independent of gender (48% in those aged >69, 95% CI = 0.36–0.77).

**Conclusion:**

Our findings suggest that there is an inverse association between schizophrenia and urinary calculi. Future studies are needed to elucidate the mechanisms by which schizophrenia negatively associates with urinary calculi.

## Introduction

It has long been observed that people with schizophrenia have higher all-cause morbidity and mortality rates when compared to the general population [1]–[3], and the gap has been widening in recent decades [4]. Two previous studies found that over half of their subjects with schizophrenia had been diagnosed with at least one medical co-morbidity [5], [6]. In another previous study with a dataset including subjects with schizophrenia or schizoaffective disorder and controls who filed at least one claim for medical services, it showed that the mean age of the cases was 40.2 years and one-third of the cases had three medical co-morbidities or more than controls [3]. Frequently reported medical co-morbidities include infections, obstetric complications, cardiovascular diseases, respiratory, endocrinologic, and metabolic illnesses, and alcohol and other drugs concurrent disorders [7], [8].

Although characterized by higher overall morbidity and mortality rates, people with schizophrenia have been demonstrated to have negative associations with various medical conditions. For example, after reviewing 14 epidemiological studies, Eaton et al. concluded that, despite their various methodological limitations, their studies conclusively proved that there was a lower than expected prevalence of rheumatoid arthritis among people with schizophrenia [9]. Additionally, Barak et al. analyzed a cohort of 3,226 people with schizophrenia, and observed a standardized incidence ratio (SIR) of 58% (p<0.05) for all cancers in the cohort was observed, compared to the rates in the general population [10].

Although a possible association exists between schizophrenia, polydipsia [11], [12] and urinary retention [13], little attention has been given to urinary diseases further downstream. Urinary systems might be more vulnerable among people with schizophrenia [14]–[16], urinary calculi, as one of the most frequently reported diseases of the urinary system and its detrimental consequences, requires more investigation.

A lifetime risk of urolithiasis exceeded 12% in males and 6% in females [17], [18]. Rising trends have been suggested for both the prevalence and incidence of urinary stones [17]. Furthermore, if left untreated, about 30–40% of patients develop another stone within five years of the initial episode [19], [20]. Whereas, with proper intervention, recurrence rates have been reported to be reduced up to 85% in randomized trials [21], [22]. This indicates that there is a need for the prompt detection of urinary calculi to ameliorate symptoms, decrease reoccurrence rates, and reduce healthcare costs. Assessment of the risk of urolithiasis is critical in the case of vulnerable populations, such as schizophrenia, in order to draw appropriate clinical attention and provide proper health care, if there is any relationship identified. Nevertheless, no direct evidence to date has been reported on the link between schizophrenia and urinary calculi.

In pursuit of these goals, this study utilized a population-based case-control study design to investigate the possibility of an association between schizophrenia and the occurrence of urinary calculi. The prevalence and risk of schizophrenia were examined among patients with and without urinary calculi in a claims dataset in Taiwan. Further analysis included stratification by age and sex.

## Methods

### Database

This matched, case-control study is based on a retrospective analysis of the administrative claims data of the Taiwan National Health Insurance (NHI) program. The data used in the present study were sourced from the Taiwan Longitudinal Health Insurance Database (LHID2000). The LHID2000 includes the claim data of 1,000,000 individuals randomly sampled from the 2000 Registry for Beneficiaries of the Taiwan NHI program; anyone who was a beneficiary of the NHI Program during any period in 2000 (n = 23.72 million) is included in the population used for random sampling and contains registration files, and original claim data for reimbursement. Furthermore, foreigners in Taiwan are also eligible for this program. The Taiwan National Health Research Institute, which created the LHID2000, claims that there is no significant difference in the gender distribution between the sampled enrollees in the LHID2000 and all the enrollees of the NHI program. The LHID2000 includes a principal, and up to four secondary discharge diagnosis codes (International Classification of Diseases, Ninth Revision (ICD-9-CM code)). Therefore, the LHID2000 allows researchers to trace all the medical services and diagnoses of these 1,000,000 individuals since the initiation of the NHI in 1995. Numerous researchers have used this dataset to perform and publish their studies in peer-reviewed journals.

As the LHID2000 consists of de-identified secondary data released to the public for research purposes, this study was exempted from full review by the director of the Taipei Medical University Institutional Review Board (IRB).

### Study sample

In selecting our cases, we first identified 54,762 patients newly diagnosed with urinary calculi (ICD-9-CM codes 592, 592.0, 592.1, or 592.9) during ambulatory care visits (including outpatients department of hospitals and clinics) during the period between 2002 and 2008. In efforts of mitigating the potential of including a miscoded case and increasing the validity of our case selection, we only included urinary calculi diagnoses made by certified urologists. In addition, we limited our cases to the adult population ≥18 years of age. Ultimately, there were 53,965 cases in this study. The index date for the cases was the first diagnosis of urinary calculi occurring during the study period.

We also selected 269,825 controls from the LHID2000. We matched each case with three controls based on sex, age and index year. We assigned each control an index date corresponding with their first ambulatory care utilization occurring during the index year. We also assured that no control had ever received any diagnosis of urinary calculi since the implementation of the NHI program in 1995.

### Key variables of interest

The main independent variable of this study was dichotomous: whether or not a person had been diagnosed with schizophrenia (with a diagnosis code of any ICD-9-CM 295 code other than 295.7-schizoaffective disorder) before the index date. In this study, we only included a diagnosis of schizophrenia if it either occurred in an inpatient setting, or appeared in two or more ambulatory care claims coded before the index date.

### Statistical Analysis

We used the SAS statistical package (SAS System for Windows, Version 8.2, SAS Institute Inc., Cary, NC) to perform all the analyses. We used the χ 2 test for proportions to test for differences in sociodemographic characteristics, hyperlipidemia, diabetes, hypertension, hypothyroidism, hyperthyroidism, obesity, alcohol abuse/alcohol dependence syndrome, and polydipsia between patients with and without urinary calculi. The sociodemographic characteristics included monthly income (0, NT$1–15,840, NT$15,841–25000, and ≥NT$25,001), level of urbanization of the subject's residence (ranging from ‘most urbanized’ (level 1) to ‘least urbanized’ (level 5)), and the geographic location of the patient's residence (Northern, Central, Eastern, and Southern Taiwan). We also performed conditional logistic regression analyses conditioned on age group (<30, 30–49, 50–69 and >69) and sex to examine the association of urinary calculi with schizophrenia. In addition, odds ratios (OR) and the corresponding 95% confidence intervals (CIs) for schizophrenia were estimated in the conditional logistic regression analyses separately for each sex and age category. A two-tailed p-value of <0.05 was considered statistically significant.

## Results

Of the 323,790 study sample, the mean age was 47.8 (±15.6 years); about 56% were aged under 50 years (Table 1). There were significant differences between patients with and without urinary calculi in terms of the monthly income, level of urbanization or the geographic location of the patient's residence, hyperlipidemia, diabetes, hypertension, hypothyroidism, hyperthyroidism, obesity, and polydipsia (*p*<0.001). But there is no significant difference in the distribution of alcohol abuse/alcohol dependence syndrome between cases and controls (*p* = 0.297). Patients with urinary calculi were more likely to have monthly incomes >NT$15,840 and to reside in the eastern part of Taiwan than patients without urinary calculi.

**Table 1:** Demographic characteristics of patients with urinary calculi and controls in Taiwan in the year 2002 (n = 323,790).

Variable	Patients with urinary calculi (*n = 53,965*)		Controls (*n = 269,825*)		*P* value
	Total No.	%	Total No.	%	
Sex					1.000
Male	33,575	62.2	167,875	62.2	
Female	20,390	37.8	101,950	37.8	
Age					1.000
<30	6,073	11.3	30,365	11.3	
30–49	24,197	44.8	120,985	44.8	
50–69	18,305	33.9	91,525	33.9	
>69	5,390	10.0	26,950	10.0	
Monthly Income					<0.001
NT$1–15,840	19,528	36.2	116,360	43.1	
NT$15,841–25,000	21,977	40.7	95,430	35.4	
≥NT$25,001	12,460	23.1	58,035	21.5	
Urbanization Level					<0.001
1	16,001	29.7	82,435	30.6	
2	15,584	28.9	80,110	29.7	
3	9,465	17.5	46,760	17.3	
4	7,276	13.5	34,565	12.8	
5	5,639	10.4	25,955	9.6	
Geographical Region					<0.001
Northern	25,739	47.7	130,075	48.2	
Central	14,004	26.0	62,300	23.1	
Southern	12,802	23.7	71,265	26.4	
Eastern	1,420	2.6	6,185	2.3	
Hyperlipidemia	12,196	22.6	40,204	14.9	<0.001
Diabetes	7,987	14.8	28,332	10.5	<0.001
Hypertension	16,945	31.4	59,362	22.0	<0.001
Hypothyroidism	432	0.8	1,619	0.6	<0.001
Hyperthyroidism	1,241	2.3	4,857	1.8	<0.001
Obesity	486	0.9	1,619	0.6	<0.001
Alcohol abuse/alcohol dependence syndrome	270	0.5	1,079	0.4	0.297
Polydipsia	432	0.8	1,619	0.6	<0.001

The prevalence of prior schizophrenia in patients and controls is shown in Table 2. A total of 3,119 (1.0%) subjects had been diagnosed with schizophrenia prior to the index date. This included 0.7% of the patients with urinary calculi, and 1.0% of the controls. The conditional logistic regression analysis, conditioned on patient sex and age group, indicated that the odds of having previously been diagnosed with schizophrenia for patients with urinary calculi were 0.69 (95% CI = 0.62–0.76, *p*<0.001) when compared with controls after adjusting for patient's monthly income, and level of urbanization, and the geographic location of the patient's residence, hyperlipidemia, diabetes, hypertension, hypothyroidism, hyperthyroidism, obesity, alcohol abuse/alcohol dependence syndrome, and polydipsia.

**Table 2:** Crude and adjusted odds ratios for urinary calculi stratified by the presence/absence of schizophrenia among the sampled patients.

Outcome variable	Total sample N = 323,790		Patients with urinary calculi N = 53,965		Controls N = 269,825	
	No.	%	No.	%	No.	%
Presence of prior-schizophrenia						
Yes	3,119	1.0	374	0.7	2,745	1.0
No	320,671	99.0	53,591	99.3	267,080	99.0
Crude OR (95% CI)	–		0.68*** (0.61–0.76)		1.00	
Adjusted OR^a^ (95% CI)	–		0.69*** (0.62–0.76)		1.00	

*Notes:*

a Conditional logistic regression (conditioned on sex and age) was performed to adjust for monthly income and urbanization level and the geographic location of the patient's residence, hyperlipidemia, diabetes, hypertension, obesity, hypothyroidism, hyperthyroidism, polydipsia, and alcohol abuse/alcohol dependence syndrome;

*** indicates *p*<0.001.

Table 3 presents the association of urinary calculi with schizophrenia stratified by sex. Among the male and female cases, patients with urinary calculi were 38% (95% CI = 0.55–0.71, p<0.001) and 20% (95% CI = 0.69–0.94, p<0.01), respectively, less likely to have a prior schizophrenia diagnosis as patients without urinary calculi.

**Table 3:** Odds ratios for schizophrenia among patients with urinary calculi and controls, by sex.

Presence of prior-schizophrenia	Sex			
	Male		Female	
	Patients with urinary calculi *n*, %	Controls *n*, %	Patients with urinary calculi *n*, %	Controls *n*, %
Yes	225 (0.7)	1,840 (1.1)	149 (0.7)	905 (0.9)
No	33,350 (99.3)	166,035 (98.9)	20,241 (99.3)	101,045 (99.1)
Crude OR (95% CI)	0.61*** (0.53–0.70)	1.00	0.82* (0.69–0.98)	1.00
Adjusted OR^a^ (95% CI)	0.62*** (0.55–0.71)	1.00	0.80** (0.69–0.94)	1.00

*Notes:*

a Conditional logistic regression (conditioned on sex and age) was performed to adjust for monthly income and urbanization level and the geographic location of the patient's residence, hyperlipidemia, diabetes, hypertension, obesity, hypothyroidism, hyperthyroidism, polydipsia, and alcohol abuse/alcohol dependence syndrome;

*** indicates *p*<0.001.

** indicates *p*<0.01;

* indicates *p*<0.05.

Table 4 presents the ORs for prior schizophrenia stratified by age group. Apart from the <30 group, when compared with controls, patients with urinary calculi were consistently less likely to have prior schizophrenia across all the age groups. The adjusted OR for prior schizophrenia for the <30 age group was 1.91 (95% CI = 1.45–2.53, *p*<0.001) times that of patients with urinary calculi than of controls.

**Table 4:** Odds ratios for urinary calculi among patients with schizophrenia and comparison group, by age group.

Presence of prior-schizophrenia	Age group							
	<30		30–49		50–69		>69	
	Patients with urinary calculi *n*, %	Controls *n*, %	Patients with urinary calculi *n*, %	Controls *n*, %	Patients with urinary calculi *n*, %	Controls *n*, %	Patients with urinary calculi *n*, %	Controls *n*, %
Total	61 (1.04)	160 (0.50)	195 (0.77)	1,555 (1.29)	95 (0.52)	825 (0.90)	23 (0.45)	205 (0.80)
Adjusted OR^a^ (95% CI)	1.91*** (1.45–2.53)	1.00	0.58*** (0.51–0.67)	1.00	0.58*** (0.48–0.70)	1.00	0.52*** (0.36–0.77)	1.00
Male	38 (1.02)	117 (0.58)	135 (0.81)	1,135 (1.42)	38 (0.36)	460 (0.88)	15 (0.48)	122 (0.80)
Adjusted OR^a^ (95% CI)	1.64** (1.16–2.31)	1.00	0.58*** (0.49–0.68)	1.00	0.41*** (0.31–0.56)	1.00	0.45*** (0.28–0.74)	1.00
Female	23 (1.07)	43 (0.37)	60 (0.70)	420 (1.03)	57 (0.74)	365 (0.94)	8 (0.41)	83 (0.79)
Adjusted OR^a^ (95% CI)	2.65*** (1.63–4.31)	1.00	0.60*** (0.46–0.77)	1.00	0.75* (0.58–0.96)	1.00	0.56 (0.30–1.05)	1.00

*Notes:*

a Conditional logistic regression (conditioned on sex and age) was performed to adjust for monthly income and urbanization level and the geographic location of the patient's residence, hyperlipidemia, diabetes, hypertension, obesity, hypothyroidism, hyperthyroidism, polydipsia, and alcohol abuse/alcohol dependence syndrome;

*** indicates *p*<0.001.

** indicates *p*<0.01;

* indicates *p*<0.05.

## Discussion

To the best of our knowledge, this is the first study to identify an association between schizophrenia and urinary calculi. Our results showed that a prior diagnosis of schizophrenia was associated with a 30% decrease in the occurrence of urinary calculi, after adjusting for monthly income, urbanization level, and the geographic location of the patient's residence. Males (36%) exhibited an even further reduced risk than females (17%). The most pronounced difference was found among older age groups (a reduction of 47% in those aged >69, 42% in those aged 50–69, and 35% in those aged 30–49). Conversely, a previous diagnosis of schizophrenia increased the risk of urinary calculi by 92% for patients aged less than 30 years after adjusting for potential confounding effects.

A number of reports have suggested that people with schizophrenia have higher than expected rates of some physical co-morbidities [1]–[3], while lower than expected rates of other physical illnesses [9], [10]. An array of frequently reported medical co-morbidities in schizophrenia included cardiovascular, neurological, genitourinary, respiratory, and gastrointestinal diseases [7], [23]. Whereas, people with schizophrenia were also found to possess a reduced risk for developing either rheumatoid arthritis or lung cancer [24]. Our study suggested that urinary calculi as another disease that might be negatively associated with schizophrenia.

It is worth noting that the risk reduction in urinary calculi was more remarkable among males than females. Sex hormones may be involved in the gender differences [25], though future studies are needed to further elucidate the reasons.

The reduced risks were more apparent among elder patients than younger ones. While the rationale for the trend of decreased risk is as yet unknown, some possibilities might be suggested. Renal dysfunction experienced in those with undiagnosed urinary calculi due to schizophrenia (via possible decrease in pain level or help seeking tendency) could be responsible for a disproportional increase in mortality. Consequently, a disproportionately small number of those with schizophrenia and urinary calculi could be observed in the older age groups. Furthermore, although most psychotropic medications are not metabolized or excreted by the kidneys, a large number of medications taken by those with schizophrenia could potentially exacerbate renal dysfunction. Also given that diabetes and hypertension are highly comorbid with schizophrenia [7], their combination with the renal dysfunction caused by urinary calculi could be particularly detrimental. It is also possible that people at a younger age were less affected by potential confounders (e.g., living conditions, dietary habits, history of previous medication). More studies should be devoted to explore the role that age plays in the link between schizophrenia and urinary calculi.

The mechanism of the negative relationship between schizophrenia and urinary calculi is unknown; yet preliminary evidence suggests several potential explanations. First, thyroid dysfunction, particularly hypothyroidism, may be contributory. Existing literature suggests that primary hyperparathyroidism or hyperthyroidism were conditions that may increase the risk of calcium stone formation [26]–[28]. However, hypothyroidism was found to be nearly three times as prevalent among people with schizophrenia than among non- schizophrenia people [3], [7]. Hypothyroidism might cause the risk of stone formation to subsequently decrease and deserved further investigation.

In addition, although speculative, as coffee, tea, beer and wine are associated with a decreased risk of stone formation [29], higher than average rates of alcohol use and caffeine consumption among people with schizophrenia might contribute to their reduced risks in urinary calculi [23].

Finally, many co-morbid illnesses may be undiagnosed or misdiagnosed in people with schizophrenia [7], [23]. Barriers to care delivery, such as the patients' poor ability to recognize medical conditions and disinclination to seek medical help, might cause healthcare professionals to overlook co-existing medical problems. All the above factors may contribute to the reduced risk of being diagnosed with urinary calculi among these vulnerable individuals.

An important strength of our study is that it utilized a large case-control population-based design, leaving little room for selection and non-response biases. By utilizing a claims dataset, this study largely avoided recall bias, a major concern in case-control studies in general.

However, the findings of this study need to be interpreted within the context of three limitations. First, as stone disease is rarely fatal and is characterized by variable pain intensity, calculi may often prove asymptomatic in the absence of obstruction. The diagnosis of urinary calculi in the claims dataset of our study was thus subject to a patient's individual pain tolerance and healthcare utilization behavior. Second, the social stigma associated with schizophrenia and the lack of insight into schizophrenia from seeking medical attention might prohibit patients with psychosis from receiving an appropriate diagnosis or treatment, which would contribute to a possible misclassification bias. Nevertheless, schizophrenia is classified as a major catastrophic illness under the National Health Insurance scheme. In Taiwan, once the diagnosis of a major catastrophic illness has been verified, the co-payment for all subsequent outpatient and inpatient healthcare utilizations is waived. It is thus in the patients' best interest that schizophrenia is faithfully diagnosed and appropriately treated. To further increase the diagnostic validity of schizophrenia in this study, we also confirmed that a prior diagnosis of schizophrenia could only be included if it was received in an inpatient setting, or appeared in at least two ambulatory care claims coded before the index date. Third, some important variables such as a family history of stone disease [30] and the patient's body mass index [31] and diabetes status [32], which are likely risk factors of urinary calculi, were unavailable in our LHID2000 claims dataset. Therefore, our findings could be compromised. Finally, the study population mainly consisted of Taiwanese Chinese, and therefore, the results may lack generalizability to other ethnic populations.

In sum, this is a pioneering study in exploring the association between schizophrenia and urinary calculi. Replication of our findings is warranted in other regions or countries. More studies will be needed to examine whether the apparent reduction in the risk of urinary calculi among schizophrenia is fictitious, and merely an artifact of under-diagnosis, or if it reflects a true decreased risk of urolithiasis among people with schizophrenia.Thus, if under-diagnosis is an issue, dissemination of our negative findings to healthcare professionals may raise awareness and help compensate for a possible deficit of medical attention paid on the urinary health of patients with schizophrenia. Prolonged hospitalizations, treatment failure, and decreased quality of life due to under-diagnosed may thus be avoided. Future studies are needed to elucidate the mechanisms by which schizophrenia negatively associates with urinary calculi.
